# A Comprehensive Review of Occupational Medicine in the Paint Industry: Roles of Family Medicine and Internal Medicine

**DOI:** 10.7759/cureus.76970

**Published:** 2025-01-05

**Authors:** Joshua A Jogie

**Affiliations:** 1 Faculty of Medical Sciences, The University of the West Indies, St. Augustine, TTO

**Keywords:** family medicine, internal medicine, occupational hazards, paint industry, worker health

## Abstract

This review explains how chemical and physical workplace hazards affect paint workers. It includes common risks like solvent exposure, heavy metals, dust, and ergonomic strain. Primary care physicians can identify early symptoms and guide primary prevention efforts. Internal medicine specialists can manage complex cases and chronic conditions linked to toxic exposures. This review shows how coordinated care and regular monitoring can reduce harm. It also explains how clinical screening, vaccination, and health education can help protect workers. By bringing together occupational medicine insights with family and internal medicine approaches, employers and clinicians can promote a safer environment for paint workers. Data for this review was gathered by searching the literature for peer-reviewed articles, official guidelines, and regulatory documents.

## Introduction and background

The paint industry employs a large workforce that handles raw chemicals, pigments, and solvents. Workers often face acute and chronic risks to their skin, eyes, and respiratory systems. Many also face ergonomic issues from heavy lifting and repetitive motions. Paint formulators combine chemical ingredients to achieve specific properties such as color, viscosity, and drying time. These tasks can lead to inhalation of volatile organic compounds (VOCs), exposure to heavy metals like lead and chromium, and contact with potential skin irritants [1]. In many countries, newer regulations limit certain toxins, but many processes still pose risks if employers do not follow safety guidelines [2]. Studies show that small-scale operations may lack adequate ventilation systems, protective equipment, or screening programs. This gap leaves workers vulnerable to illnesses that may present in subtle ways [3].

The paint industry poses many challenges that require ongoing review. Workers handle solvents, pigments, resins, and other chemicals that can cause acute and chronic harm. Many factories adopt protective measures, but gaps remain in smaller or understaffed facilities. Regulators try to reduce risks through guidelines, yet these guidelines may not be understood or followed.

Primary care physicians (PCPs) and internal medicine specialists see patients who work in a variety of industries, including paint production and application. These clinicians see patients who present with issues linked to paint exposures and often manage early signs of work-related conditions, which can appear as nonspecific symptoms like headaches, dizziness, or rashes [4]. Symptoms related to paint exposure can mirror common ailments such as viral infections or stress-related complaints. Without an occupational history, these conditions might be misdiagnosed or attributed to lifestyle factors. This misinterpretation can delay proper intervention, leading to chronic health problems [5]. Occupational medicine practitioners collaborate with family medicine and internal medicine physicians to identify chemical exposures, design prevention strategies, and provide early intervention.

The paint industry offers a useful example of how chemical, physical, and psychosocial hazards intersect. The collaboration among occupational health professionals, employers, and primary and specialty care providers continues to evolve. Addressing the risks requires not just specialized occupational medicine input but also active involvement from family and internal medicine practices. The objective of this review is to show how primary care and internal medicine can integrate occupational health strategies to protect paint workers from chemical and physical hazards. It will detail common exposures, discuss early detection methods, and outline collaborative preventive measures. By clarifying each specialty’s role, the review aims to promote coordinated care and reduce paint-related illnesses. 

## Review

Risks in the paint industry and the role of family medicine and internal medicine specialists

In the paint industry, VOCs can irritate the eyes and respiratory tract, cause dizziness, and lead to fatigue [6]. Chronic exposure can harm the liver and kidneys. Some compounds may also act as sensitizers, causing asthma or contact dermatitis. Workers applying paints may not know how to read safety data sheets. They might not realize that consistent exposure over the years can affect their health. Many do not wear protective masks or gloves if employers fail to provide them or enforce their use [7].

Metal-based pigments can introduce other hazards. Lead-based pigments were common in older paints, so workers who deal with historical building renovations may still encounter them [8]. Lead exposure can cause anemia, neurological symptoms, and decreased kidney function. Chromium, often used in pigments, can induce allergic contact dermatitis, skin ulcers, or respiratory irritation [9]. Cadmium and cobalt are sometimes present too. PCPs might see mild cases of toxicity that present as generalized weakness or gastrointestinal distress. Internal medicine specialists might see advanced organ damage or complex multisystem involvement [10].

Solvent abuse or misuse can happen when workers use industrial solvents in unventilated rooms. Toluene, xylene, and other solvents can cause acute intoxication, impacting coordination and mental status [11]. Chronic inhalation might lead to memory issues and neurological changes. The paint industry also uses isocyanates in some polyurethane products, which can cause occupational asthma. Family medicine providers can detect specific breathing problems, such as tachypnea or shortness of breath, during routine checkups. Internal medicine physicians can manage more advanced respiratory or immunologic conditions [12].

Protective measures include personal protective equipment (PPE) like gloves, respirators, and safety goggles. Workers should have access to washing stations, adequate ventilation, and training on safe handling procedures. But in smaller workshops, these measures may be overlooked. In large-scale industrial settings, strict protocols and regular audits are more common, yet lapses can still occur if workers become complacent or if management reduces safety expenditures [13].

One challenge is that workers often do not recognize the long-term implications of exposure to paint-related toxins. Family medicine physicians can educate patients about the importance of consistent PPE use. They can also conduct periodic lung function tests, skin exams, and neurological assessments. Internal medicine specialists can perform more specialized evaluations, especially in cases where organ function might be compromised [14].

Ergonomic risks in the paint industry can lead to musculoskeletal disorders. Workers may lift large containers or remain in awkward positions while spraying. Back pain and repetitive strain injuries can result. Simple interventions like adjustable workstations, rotating tasks, and ergonomic training help minimize these issues [15]. PCPs might see patients reporting musculoskeletal complaints that have unclear etiology. A thorough occupational history can guide effective treatment plans. Internal medicine physicians might address more severe cases requiring surgery or physical therapy [16].

The paint industry’s supply chain also involves transportation and storage. Workers moving large containers can face additional hazards. Chemical leaks can occur if containers are not sealed properly or if workers lack proper knowledge of chemical compatibility. Such leaks can pose acute dangers to exposed individuals and the environment [17]. Family and internal medicine physicians might be called upon to treat chemical burns or acute systemic toxicity if an accident occurs. Timely intervention can reduce morbidity and mortality [18].

Vaccination programs can help protect against certain infectious agents that may complicate underlying conditions. For example, influenza vaccination can help workers with compromised respiratory systems. Tetanus shots are also critical, as many workers handle sharp metal objects, and cuts may occur in industrial settings [19]. Education campaigns are useful to raise awareness about the importance of these vaccinations. Family medicine clinics often serve as a primary point for vaccine administration, and internal medicine specialists coordinate with PCPs for patients who have comorbid conditions [20].

Preventive screening is vital. Annual exams and blood tests can detect early signs of heavy metal exposure. Spirometry can identify subtle changes in lung function, especially for those handling isocyanates or working in poorly ventilated environments [21]. Hearing tests can also be useful if machinery noise is high. These tools help clinicians track any downward trends in health. Early detection can lead to job modifications or improved protective measures. Internal medicine physicians can further explore complex pathology when basic screening flags an issue [22].

Monitoring programs can become part of a broader occupational health strategy. When workers undergo regular checkups, they become more engaged in their own health. They may also receive training on risk recognition, first aid, and emergency response. Education on chemical labeling, protective gear use, and safe disposal practices can reduce injuries. PCPs can reinforce this education during patient visits. Internal medicine specialists can show how certain diseases might worsen without adherence to safety protocols [23].

Collaboration between employers, safety officers, and clinical teams can streamline these efforts. Some workplaces hire or contract occupational health experts who design safety guidelines and perform on-site inspections. However, in some areas or smaller facilities, this role is filled by a PCP or an internist who has an interest in occupational health. They may conduct periodic visits to the plant or workshop, checking for compliance and updating guidelines based on new research [24].

There can be psychosocial factors too. Paint industry workers may face job insecurity, language barriers, or limited access to healthcare. This environment can make it hard to report symptoms or request safer working conditions. PCPs often serve as advocates by communicating with employers or public health authorities. Internal medicine physicians can write detailed reports that document work-related illnesses, which can influence policy changes. When these roles align, the workforce benefits from more coordinated care [25].

Occupational stress can impact mental health. People who work long hours with tight deadlines for paint production or application may develop anxiety, fatigue, or depression. Solvent exposure can cause mood changes too. PCPs may see these symptoms in primary care settings. Internal medicine experts might manage more severe mental health conditions, sometimes in collaboration with psychiatrists. Early intervention can improve adherence to safety protocols because workers are more inclined to protect themselves when they feel supported and healthy [26].

Research shows that effective hazard communication, the use of less toxic materials, and the enforcement of safety regulations can reduce workplace injuries. However, progress can be slow when industries resist changes that increase costs [27]. In areas with less stringent legislation, workers are more at risk. Family and internal medicine physicians can help highlight these risks by publishing case studies or reaching out to community organizations. They can encourage more stringent inspections and advocate for best practices. This approach extends beyond individual treatment to population-level health [28].

For instance, water-based paints have fewer VOCs; however, they are not always favored by contractors who are used to solvent-based alternatives. Education at the primary care level can show workers and employers the potential long-term health benefits of adopting water-based solutions [29]. Internal medicine specialists might see data on how inhalation of certain solvents correlates with neurological outcomes over decades. They can share this evidence to drive safer material selection. Family doctors might present simpler messages to their patients about the immediate and future health benefits of safer paints [30].

Changes in the paint industry have produced more eco-friendly formulations. This shift helps the environment and may reduce certain health hazards. Still, new chemicals can pose different risks that need further study. A multipronged approach that includes epidemiological research, laboratory testing, and clinical observation is vital to keep up with evolving paint components [31]. Family and internal medicine physicians can take part in these efforts by reporting adverse effects they see in practice. When combined with occupational health data, these reports guide better safety standards.

Chronic conditions like hypertension or diabetes can complicate occupational exposure. Workers with existing comorbidities may need closer monitoring. Certain toxins may worsen renal function or cardiovascular status. PCPs can adjust medications or advise more frequent follow-ups. Internal medicine specialists may offer advanced interventions to manage end-organ damage when exposure persists. This interplay underscores how occupational health integrates with regular medical care [32].

In many settings, paint industry workers might live in crowded housing, sometimes near industrial sites with environmental pollution. This scenario creates multiple exposure pathways. Family medicine visits are often the first place these social and environmental issues are noticed. Physicians can connect families with social services or public health programs, reducing overall health risks [33]. Internal medicine physicians can confirm diagnoses of toxic exposure through specialized tests or imaging. This collaboration helps close the loop, providing holistic care.

Educational programs for medical students and residents can reinforce the importance of occupational history-taking. Family and internal medicine residency curricula should include modules on common industrial exposures, such as paints and solvents. Clinical simulations can highlight how to recognize subtle signs of toxicity. This training fosters a new generation of physicians who can blend clinical knowledge with occupational health principles [34].

Certain groups may be more at risk. Younger workers might be unaware of safety protocols or consider themselves invulnerable. Older workers might already have reduced pulmonary function or pre-existing chronic diseases. Pregnant workers face fetal risks from toxic exposures. PCPs must address these diverse populations in routine care. Internal medicine specialists may handle high-risk pregnancies or older patients who have multiple conditions. Tailoring prevention and early detection efforts are necessary for effective health outcomes [35].

Sometimes, paint operations occur in informal settings or in individuals’ homes. In such cases, there may be minimal regulation, no ventilation, and no PPE. Home-based spray painting or paint mixing can create health risks for other household members. PCPs who practice in the community can identify these situations. They can educate families on proper ventilation, storage, and handling techniques. Internal medicine specialists can manage complications that arise from unregulated exposures, emphasizing the importance of better oversight at the policy level [36].

Occupational health specialists note that ongoing research can improve understanding of emerging paint additives. Nanoparticles may be used to enhance paint properties. Their health effects are not fully known. Animal studies suggest potential respiratory issues, while human data remains limited [37]. Family and internal medicine physicians can keep updated on new developments through continuous medical education. Reporting any suspicious health pattern to occupational authorities or researchers can alert policymakers and the broader scientific community. This feedback loop helps shape safer regulations [38].

Table 1 summarizes the main chemical exposures, their potential health effects, and recommended safety measures.

**Table 1:** Common Chemical Exposures in the Paint Industry, Their Effects, and Safety Measures

Chemical Exposure	Potential Effects	Safety Measures
Solvents (e.g., xylene)	Headache, dizziness, skin irritation, liver or kidney damage	Use respirators, wear gloves, ensure proper ventilation, and follow safe handling protocols
Heavy Metals (e.g., lead)	Neurological damage, anemia, organ dysfunction	Regular blood tests, strict handling procedures, and consistent PPE use
Isocyanates (in polyurethane paints)	Occupational asthma, skin inflammation, eye irritation	Closed mixing systems, local exhaust ventilation, personal respirators
Volatile Organic Compounds (VOCs)	Respiratory irritation, headache, potential chronic lung damage	Install advanced ventilation systems, limit exposure time, wear appropriate masks
Pigments and Resins	Allergic reactions, potential long-term respiratory problems	Use enclosed mixing stations, follow SDS guidelines, and rotate tasks to avoid prolonged exposure

Some safety measures and monitoring

Economic factors influence how well safety measures are implemented. Some workplaces fund robust occupational health programs, complete with on-site clinics, frequent training, and advanced ventilation systems. Others may cut costs, resulting in less protection for workers [39]. PCPs and internists who serve these workers can provide evidence that investing in safety reduces long-term healthcare costs. Studies show that early intervention decreases sick days, boosts productivity, and lowers compensation claims [40]. This data can persuade employers to prioritize safety.

Regular Screening

Researchers have examined how regular screening can detect early lung function declines in paint workers. Some studies show that spirometry can pick up small abnormalities before they become symptomatic. These findings align with broader evidence showing that early detection can reduce long-term costs. Workers who receive a prompt diagnosis of occupational asthma, for example, can limit further exposure or switch tasks [41]. But screening alone does not suffice. Without proper guidance, workers might ignore early signs and continue working under the same conditions. PCPs can reinforce the importance of follow-up visits, and internal medicine specialists can dig deeper into organ function if blood tests or imaging reveal concerning trends. 

Biological Monitoring

Biological monitoring for heavy metals has also proven valuable. Over time, paint workers may accumulate lead, chromium, or other metals in their bodies. Repeated monitoring can show whether any new safety measure is effective, and it can help identify unnoticed leaks or poor work practices [42]. In workplaces that switch to low-lead or lead-free materials, blood lead levels can drop in a matter of months. However, these improvements depend on strict handling procedures and consistent use of PPE. PCPs can interpret these monitoring results for workers, while internal medicine experts can address organ damage if it emerges.

Ergonomic Considerations

Ergonomic considerations matter as well. Many reviews point out that repetitive motions in paint application can strain the shoulder, wrist, or lower back [43]. Proper training on spray-gun handling, along with rotating tasks, can prevent chronic musculoskeletal disorders. When clinicians see patients who complain of persistent joint pain, an occupational history can pinpoint how their daily work patterns contribute. Some studies recommend on-site physiotherapy sessions or group education events. These interventions have lowered absenteeism in certain plants [44]. A combined approach from family medicine, internal medicine, and occupational health can keep mild cases from worsening.

Ventilation Systems

Some factories have embraced advanced ventilation systems, such as local exhaust hoods near mixing stations. Research shows that these solutions can significantly lower ambient levels of VOCs [45]. Yet cost remains a concern. Smaller businesses sometimes rely on open windows or fans, which may not capture enough fumes. Advocates suggest government subsidies for improved engineering controls. PCPs observing a high incidence of respiratory issues in a local workforce can alert public health officials. Internal medicine professionals can share case data on hospitalized patients to bolster claims about inadequate controls. This teamwork has influenced policy changes in regions where paint manufacturing is prevalent.

Labeling

Proper labeling and chemical safety data sheets (SDS) are also crucial. Workers often do not read or fully understand these sheets if they are complex or written in a language they do not speak well [46]. Some solutions include translating SDS into local dialects or creating simplified summaries with icons. Clear labeling helps the entire healthcare team too, because clinicians can quickly identify the agents in question if a worker presents with acute toxicity. Reference materials remain vital in emergency cases, where immediate knowledge of the chemical can inform the choice of treatment. However, labeling alone will not work if workers lack basic education on chemical properties.

Education

Educational programs in paint factories can increase compliance and foster a safety culture. These programs cover topics like the proper mixing ratios, safe storage, and recommended PPE for each chemical. Some research shows that interactive sessions, such as workshops led by occupational health nurses, can make a lasting impression [47]. Workers are more likely to wear gloves or goggles when they understand the rationale and see real-life examples of the consequences of noncompliance. Family and internal medicine physicians can reinforce these teachings in their clinics. If a patient arrives with a minor chemical burn, a physician can review the importance of protective gear and the correct first-aid steps to ensure it does not happen again.

Waste Disposal

Another area of concern is the disposal of waste materials. Improper disposal can contaminate soil and water sources. Communities located near dumping sites may experience increased rates of disease linked to long-term low-dose exposure [48]. PCPs practicing in these communities might see clusters of unexplained symptoms, prompting investigations. Internal medicine physicians can contribute data on organ involvement or severity patterns. Some researchers call for better tracking of waste disposal routes and heavier fines for illegal dumping. Collaboration with environmental agencies can reduce broader health risks, especially for vulnerable populations like children or older adults.

Mental Health Checkups

The role of psychological stress in the paint industry is receiving more attention. Tight deadlines, shift work, and the fear of chemical exposure can weigh on workers. Chronic stress can worsen physical outcomes, reducing immune function and making individuals more susceptible to disease [49]. Mentally strained workers might neglect safety steps or skip medical checkups. Studies report that counseling services, peer support groups, and stress management training can improve overall productivity and decrease accidents [50]. PCPs can screen for depression or anxiety, and internal medicine physicians can watch for psychosomatic presentations or stress-related disorders. When combined with occupational medicine strategies, mental health support can further reduce workplace incidents.

Evaluation of Changes

Changes in paint formulations continue. Researchers explore ways to reduce VOC content and minimize heavy metals. Water-based coatings have become more common, yet some lines still rely on solvents for specific finishes. Innovative manufacturers experiment with biobased solvents made from agricultural by-products [51]. Though these new products may offer lower toxicity, they require thorough evaluation. Lab tests and small-scale trials might not capture the full range of hazards that appear when thousands of workers handle these products daily. Ongoing reviews of clinical data can show any unexpected patterns. PCPs could notice new dermatological reactions or respiratory complaints, while internists might detect rare complications among hospitalized patients.

Asthma Checks

Isocyanates, used in many polyurethane paints, remain a major cause of occupational asthma. Researchers note that even short-term exposure can sensitize workers, leading to severe symptoms [52]. Proper ventilation and closed mixing systems help, but personal respirators and routine air monitoring are also key. Some factories have replaced isocyanate-based systems with alternatives, but these may have their own risks. Occupational medicine professionals must balance the benefits of each formulation. PCPs and internal medicine specialists track outcomes over time. If new-onset asthma cases decrease, that suggests the replacement worked, at least in terms of respiratory hazards.

Hearing Checks

Noise control rarely gets the same attention, but it is relevant. Certain paint production processes involve high-speed mixers or grinders. Prolonged noise exposure can cause hearing loss. Workers who also face solvent exposures might experience combined effects on the auditory system [53]. PCPs can identify early signs, especially if patients complain about trouble hearing conversations in noisy environments. Internal medicine specialists can investigate more complex auditory or neurological symptoms. Noise-induced hearing loss is irreversible, so prevention is crucial. Ear protection and periodic hearing tests are standard recommendations.

Changing Work Clothes

Indoor air quality is another concern, especially in enclosed spray booths. Some studies show that even if workers wear respirators, residual airborne chemicals can settle on clothes or hair and be inhaled later [54]. Best practices involve dedicated changing areas, daily uniform laundry, and sometimes disposable coveralls. Workers should also avoid bringing contaminated clothing home, where family members could be exposed. Family and internal medicine physicians treating individuals with nonspecific symptoms should ask about the possibility of secondary exposures at home. This approach can reveal a hidden link to workplace toxins.

Coordination between the different medical specialties

Coordination among different medical specialties can be formalized through occupational health networks. Some regions have set up electronic databases that log each worker’s exposure profile, medical history, and lab results. When a patient visits a pCP, that physician can quickly see the relevant workplace hazards. If the case escalates, an internal medicine physician can pick up where the family doctor left off, without duplicating tests [55]. This continuity speeds up care and helps researchers gather epidemiological data. Some networks expand beyond the paint industry, but the principle is the same: connect health data across different providers to catch patterns early.

Community engagement can strengthen these efforts. Workshops in which workers, employers, public health officials, and clinicians meet can bridge communication gaps. Family and internal medicine physicians who attend these meetings gain insights into the daily challenges that paint workers face, from insufficient rest breaks to high production quotas. Employers learn how simple modifications in scheduling or rest area design could reduce stress and potential accidents [56]. Public health officials gather real stories that inform better policies. Occupational medicine specialists present statistical trends or best practices, guiding the group on the next steps. Such collaborative forums have led to lower accident rates and higher worker satisfaction in certain pilot programs.

Research also points to the need for mental health resources geared specifically to industrial labor. Many paint workers migrate for these jobs, leaving family support systems behind. They may live in dormitories or shared housing near factories, with limited recreational space. Lack of social support may intensify stress or lead to unhealthy coping mechanisms like substance abuse [57]. PCPs who notice these patterns can coordinate with local mental health counselors. Internal medicine specialists might deal with advanced alcohol-related liver disease or other complications. Occupational health teams can work with management to create better living conditions and more recreational activities. These measures improve morale and reduce safety violations.

Pre-employment health evaluations help identify workers who have preexisting conditions that could be worsened by paint exposure. For example, someone with uncontrolled asthma might be at higher risk in a job that handles isocyanates. A basic physical exam, spirometry, and relevant blood tests can screen applicants. This process helps employers place workers in suitable roles or institute extra precautions [58]. PCPs are often part of these exams in smaller communities. Internal medicine specialists may step in if a prospective employee has a more complex medical history. This early identification prevents future harm and reduces turnover. It also underscores the importance of a comprehensive approach.

Protecting pregnant workers in the paint industry requires special attention. Many solvents and metals can affect fetal development. Some chemicals cross the placenta or accumulate in breast milk. Employers should limit exposure through job restructuring or by allowing pregnant workers to shift to tasks with fewer risks [59]. Family medicine physicians typically provide prenatal care and can advise on safer work practices. Internal medicine physicians may need to evaluate pregnant workers with underlying health issues, such as hypertension or gestational diabetes, in combination with exposure risks. This joint effort aims to protect both the mother and the fetus.

The paint industry relies on supply chains that extend to transport, packaging, and retail. Drivers hauling chemical loads face their own risks from fume leaks or accidents. Retail workers sometimes manage paint mixing stations without robust ventilation. Occupational medicine extends to these peripheral roles. Family and internal medicine physicians may see employees from every link in that chain. They can ask routine questions about job tasks, chemical handling, and safety measures. Documenting these exposures can help identify patterns that indicate a need for industry-wide changes [60]. For instance, if multiple patients from different locations show similar symptoms, it may point to a widespread issue with a particular product or process.

Clinical guidelines continue to evolve as researchers publish new findings. Some guidelines recommend annual lung function tests for workers with more than five years of solvent exposure. Others propose creating a standard threshold for metal levels in the blood, after which workers must be reassigned to a less toxic role. PCPs can help with broad testing, while internal medicine specialists interpret borderline or ambiguous results. The final decision about job suitability sometimes involves the occupational health team and the employer. There is also debate about how often these guidelines should be updated, given that paint formulations and regulatory standards are in flux.

Reviews highlight that adequate data collection is still lacking in many parts of the world. Researchers often focus on well-regulated industries in wealthy nations, leaving a gap in knowledge about smaller or informal operations in developing regions. PCPs in those areas might have limited resources, making it hard to link symptoms to paint exposure or to run specialized tests. Internal medicine physicians might see advanced cases that have been neglected for years. Collaborative research networks could address this gap by standardizing data collection and sharing results across borders. Doing so would help form a global picture of occupational risks in the paint sector and guide international guidelines and training materials.

Some researchers suggest that wearable sensors might improve hazard detection, alerting workers or supervisors when chemical concentrations exceed safe limits. Early pilot studies show promise, but cost and implementation barriers remain [41]. Family medicine clinics may not have immediate access to these technologies, though future initiatives might encourage widespread adoption. If sensors become more affordable, smaller workshops might incorporate them. Internal medicine specialists analyzing complex cases could benefit from sensor data that pinpoint the timing and intensity of exposures. Over time, real-time monitoring could reduce the guesswork about what caused a patient’s symptoms. Reliable objective data might also strengthen compensation claims if a worker is harmed by proven overexposure.

Vaccine research is ongoing for certain infections that can complicate paint-related exposures, though this remains an emerging field. Some studies look at how immunocompromised workers might face greater risks from bacterial or fungal growth on damp surfaces within painting facilities. Mold can thrive in poorly ventilated areas, and some paints promote moisture retention [42]. PCPs see a range of symptoms, from respiratory infections to allergic responses. Internal medicine specialists address more severe pulmonary complications. Integrating vaccination strategies with standard workplace safety measures could lessen these risks.

Digital tools are being tested for training. Mobile apps can illustrate safe mixing techniques, highlight toxicity levels, and show correct PPE use. Workers can access these tools in multiple languages, bridging literacy gaps. Some data indicate that interactive apps improve retention compared to traditional slideshows [43]. However, not all workers have smartphones, and connectivity can be an issue in rural areas. PCPs in those areas might rely on paper-based materials or group demonstrations instead. Internal medicine physicians who do telemedicine consults can still use digital visuals to explain test results or show basic anatomy, helping patients grasp how certain toxins affect different organs.

Roles continue to evolve as new chemicals enter the market. Some paints add antimicrobial agents, which might reduce bacterial growth on painted surfaces; however, the long-term effects of inhaling or handling these agents are not well-known. Researchers conduct in vitro studies to see if they can irritate the lungs or trigger immune responses [44]. Occupational health experts urge caution until more data emerge. PCPs can record any new patterns in local clinics, like unusual rashes or persistent coughs. Internal medicine specialists can order advanced diagnostic tests if standard treatments fail. This iterative loop of observation, testing, and policy revision keeps pace with industry changes.

Collaboration among institutions that oversee labor, health, and the environment can ensure that rules are consistent. Some countries have separate agencies that do not always share data. If a paint factory passes an environmental inspection, it might not necessarily meet labor safety standards. Streamlined oversight can reduce confusion for employers and employees [45]. PCPs can facilitate this process by encouraging patients to report any workplace hazards. Internal medicine physicians can document specific pathologies that may indicate systemic failures. Over time, a coordinated system can generate more robust statistics on occupational diseases, clarifying trends and helping set research priorities.

Social media sometimes amplifies stories of worker injuries or diseases. While this can raise public awareness, it might also spread misinformation. Occupational health professionals can use these platforms carefully to share accurate tips or direct workers to credible medical services [46]. PCPs and internists can correct misconceptions if patients arrive with exaggerated fears or suspect cures found online. They can also encourage workers to share legitimate concerns on official channels. This modern communication landscape can spark faster responses to critical incidents, such as chemical spills or sudden illness clusters.

The family medicine and internal medicine perspectives are vital because they encompass the whole patient. Occupational medicine focuses on workplace exposures, but many factors shape health. A worker might have poor nutrition or live in crowded housing that heightens susceptibility to disease. PCPs often see these broader contexts, which can worsen occupational risks. Internal medicine specialists treat advanced conditions that may be rooted in social or environmental stressors. By working together, these fields can develop more holistic plans that address both immediate workplace dangers and broader lifestyle or public health factors [47]. Such synergy can improve adherence to safety guidelines and follow-up recommendations.

Many paint workers never see an occupational health physician unless required by their employer; however, they do see family or internal medicine doctors for routine problems like high blood pressure or the flu. This makes primary and specialty care a natural gateway to detect potential occupational exposures. Asking a few targeted questions about paint handling, protective measures, or workplace safety can reveal hidden clues. If a pattern appears, like multiple patients from the same factory with similar complaints, the doctor can contact a local occupational health team or regional health authority for further investigation [48]. This approach turns ordinary clinic visits into a surveillance tool.

Some have proposed incentives for factories that show a decrease in work-related illness rates. These incentives might be lower insurance premiums or public recognition for meeting safety benchmarks [49]. Workers might also receive bonuses for consistently following safety protocols. However, these programs need careful monitoring to prevent underreporting of injuries or illnesses. Family and internal medicine clinics might detect suspiciously low rates of occupational complaints if they suspect employees fear losing bonuses. Transparent data collection and anonymous reporting channels can mitigate such risks.

In the end, the paint industry and the broader healthcare community share responsibility for worker well-being. This review underscores how family and internal medicine interact with occupational medicine to improve prevention, detection, and treatment. While the background outlined the core hazards and general strategies, these review points highlight real-world applications, newer research insights, and the evolving role of interdisciplinary collaboration. The challenge lies in addressing chemical, physical, and psychosocial factors all at once. Researchers continue to refine guidelines and explore innovative solutions, but consistent efforts from employers, workers, and clinicians remain essential for sustained progress.

## Conclusions

Workers in the paint industry face chemical and physical hazards that can cause acute and chronic illnesses. By reducing exposure and educating workers, the paint industry can protect its workforce and manage healthcare costs. Employers can add ventilation, training, and protective equipment, but cost and worker compliance can be obstacles. Routine checkups and biological monitoring spot problems before they worsen. Early detection by primary care and internal medicine specialists can reduce severe outcomes. Occupational medicine experts guide exposure assessments and prevention. Collaboration across specialties supports prompt interventions and lowers disease rates.
